# Inhibitory Effect of Phenethyl Isothiocyanate on the Adhesion and Biofilm Formation of *Staphylococcus aureus* and Application on Beef

**DOI:** 10.3390/foods13213362

**Published:** 2024-10-23

**Authors:** Xiaojing Ma, Jinle Ma, Jianan Liu, Hongshun Hao, Hongman Hou, Gongliang Zhang

**Affiliations:** 1 School of Food Science and Technology, Dalian Polytechnic University, Dalian 116034, China; xgmdzrt_ejmbcty@163.com (X.M.);; 2 Department of Inorganic Nonmetallic Material Engineering, Dalian Polytechnic University, Dalian 116034, China; 3 Liaoning Key Lab for Aquatic Processing Quality and Safety, Dalian 116034, China

**Keywords:** *Staphylococcus aureus*, phenethyl isothiocyanate, inhibition, biofilm, adhesion

## Abstract

This study aimed to explore the mechanism by which phenethyl isothiocyanate (PEITC) inhibited the adhesion and biofilm formation of *Staphylococcus aureus* (*S. aureus*). PEITC exhibited antimicrobial efficacy against *S. aureus*, demonstrating a minimum inhibition concentration (MIC) of 1 mmol/L. PEITC exerted its antibacterial effect by disrupting cell membrane integrity, and it decreased total adenosine triphosphate (ATP) production after 1 and 4 h treatment. PEITC at 0.5 mmol/L increased the level of intracellular reactive oxygen species (ROS) by 26.39% compared to control. The mature biofilm of *S. aureus* was destroyed by 86.4% after treatment with PEITC for 24 h. Adhesion tests revealed that PEITC at 0.5 mmol/L reduced 44.51% of the *S. aureus* that adhered to NCM460 cells. Furthermore, at the genetic level, PEITC significantly downregulated the related genes by 31.26% to 97.04%, including *agrB*, *agrD*, *isdA*, *ebh*, *luxS*, *fnbA*, and *icaR.* Moreover, PEITC markedly inhibited *S. aureus* proliferation in beef preserved at temperatures of 25 and 4 °C, respectively. In summary, the present study suggests that PEITC effectively inhibits the adhesion and biofilm formation of *S. aureus* by affecting the relevant genes of *S. aureus* and holds promise for microbial management in meat products.

## 1. Introduction

The proliferation of pathogenic microorganisms has emerged as a pervasive concern within contemporary contexts, encompassing both the food and medical industries [1]. *Staphylococcus aureus* (*S. aureus*), an important Gram-positive foodborne pathogen, impacts human health on a global scale [2]. The rapid proliferation of *S. aureus* during food production can have consequential implications for food quality [3], and the production of diverse toxins associated with food may pose a threat to food safety [4,5]. Furthermore, *S. aureus* can also cause multiple illnesses in the medical field [6]. The main substances that inhibit *S. aureus* are antibiotics, organic acids and essential oils [7]. However, the biofilms formed by *S. aureus* protect it from inhibitors [8]. *S. aureus* embedded within the biofilm matrix exhibits resistance to chemical disinfectants that is 10–1000 times greater than that of planktonic *S. aureus* [9], which makes complete eradication difficult and leads to *S. aureus* contamination in food processing. For instance, in dairy production, *S. aureus* can easily adhere to stainless steel surfaces and rapidly forms a biofilm that cannot be completely removed, leading to contamination of dairy products [10]. Therefore, biofilms have become crucial points for inhibiting *S. aureus*.

Not all strains of *S. aureus* produce biofilms; it is reported that 46.1% of the *S. aureus* strains isolated from hospitals were biofilm producers [11]. However, once formed, biofilms can impede antimicrobial measures, leading to food contamination and subsequent disease outbreaks. Biofilm formation by *S. aureus* is a multistep process [12]. Bacteria adhere to carriers, which is subsequently followed by the secretion of polysaccharides and proteins, culminating in the formation of biofilms [13]. The biofilms produced by *S. aureus* pose a significant risk of contamination and subsequent infection and protect *S. aureus* against antibiotic treatment [14]. To mitigate the resistance of *S. aureus* biofilms to inhibitors, the initiation of efforts toward the development of natural antimicrobials has become the focal point [15,16]. Recent studies have shown that plant-extracted components have suppressive effects on different species of bacteria, thus attracting attention as potential alternatives to conventional antibiotics [17]. Natural inhibitors act on *S. aureus* biofilms in a variety of ways, such as by inhibiting *S. aureus* extracellular polysaccharide production, disrupting mature biofilms [18] and influencing biofilm-associated genes [19]. Most studies have focused more on biofilms; however, as a vital and initial process in biofilm formation, adhesion has not been emphasized as much as biofilms [20]. The focus has been solely on adhesion to substratum surfaces such as stainless steel, glass, and plastics [21]. Reports of natural inhibitors affecting both adhesion and biofilms in *S. aureus* are limited.

According to the findings of Qian et al., artesunate simultaneously inhibited *S. aureus* biofilm production and adhesion to polyethylene plates by suppressing the accessory gene regulator (agr) system and modulating genes like fibronectin binding A (*fnbA*) [22]. A *Litsea cubeba* essential oil-loaded peptide micro/nanotubes structure (LC-EO/FNTs) reduced the ability of *S. aureus* to form biofilms by affecting the iron surface determinant A (*isdA*) gene and reduced *S. aureus* adhesion to soy products [23]. Furthermore, *Krameria lappacea* root extract (KLRE) not only effectively inhibited the generation of methicillin-resistant *S. aureus* (MRSA) biofilm, but also reduced the adhesion of MRSA to human lung carcinoma A549 cells [24]. These indicate that natural compounds are capable of suppressing *S. aureus* growth and adhesion at the genetic level, thereby reducing biofilm formation.

**Figure 1:**
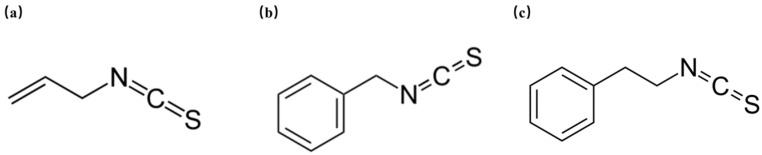
The chemical structures of AITC (**a**), BITC (**b**), and PEITC (**c**).

Isothiocyanates (ITCs) are natural compounds, found in cruciferous plants. ITCs have excellent antioxidant, anti-inflammatory, and anticancer properties and are beneficial to human health [25]. There are a wide range of ITCs, including allyl isothiocyanate (AITC), benzyl isothiocyanate (BITC), and phenethyl isothiocyanate (PEITC) (Figure 1) [26,27]. For instance, BITC was able to significantly downregulate *S. aureus* virulence genes such as thermonuclease (*nuc*), clumping factor (*clf*), and protein A (*spa*) [28]. Few studies have focused on the inhibitory effects of ITCs on biofilms of various bacteria. A mixture of ITCs was shown to significantly inhibit the biofilm formation of *Pseudomonas aeruginosa* (*P. aeruginosa*) [29], and AITC and PEITC were initially used to investigate their ability to remove biofilms of bacteria, including *S. aureus* [30]. According to previous studies, PEITC has been proven to have inhibitory effects on various bacteria, including *S. aureus*, *Escherichia coli* (*E. coli*), and *P. aeruginosa* [31]. It has been reported that auranofin synergizes with PEITC to inhibit the colonization of *S. aureus*, thereby reducing subcutaneous abscesses in mice [32]. Liu et al. also reported that inclusions formed by γ-cyclodextrin and PEITC had antibacterial effects on *S. aureus* [33]. However, the mechanism by which PEITC affects adhesion and biofilm formation, which are the key factors for *S. aureus* contamination and pathogenicity, is still unknown.

The objective of present study was to reveal the inhibitory effects and mechanisms of PEITC on *S. aureus* biofilm and adhesion. The impact of PEITC on physiological processes in *S. aureus* was explored, including cell membrane integrity, adenosine triphosphate (ATP) synthesis, reactive oxygen species (ROS) generation, and microstructure. NCM460 cells were used as hosts to assess the effect of PEITC on *S. aureus* adhesion. Furthermore, the influence of PEITC on the expression of genes related to adhesion and biofilm formation was also investigated. Finally, PEITC was applied to beef preservation, determining its inhibitory effects on *S. aureus* at 4 and 25 °C, respectively. This study provided information for further exploration of PEITC as antimicrobial reagent and potential applications.

## 2. Materials and Methods

### 2.1. Bacterial Strains and Culture Media

The *S. aureus* strain ATCC 6538 was purchased from the China Center of Industrial Culture Collection (Beijing, China). Trypticase soy broth (TSB), and agar were purchased from Hope Technology Co., Ltd. (Qingdao, China). The strains were cultured in TSB at 37 °C with shaking at 150 rpm. NCM460 cells were obtained from Stem recell (Shanghai, China). DMEM medium, fetal bovine serum (FBS) and penicillin-streptomycin (Pen-Strep) were purchased from Thermo Fisher Scientific (Shanghai, China). PEITC (purity > 99%) was purchased from Sigma (Saint Louis, MO, USA). The RNA kit DP430 was purchased from Tiangen Biotech (Beijing, China), the PrimeScriptTM RT kit with gDNA eraser was from Takara (Beijing, China), and the SYBR Green Premix Pro Taq HS qPCR Kit was from Accurate Biotechnology (Changsha, China).

### 2.2. MIC and Growth Curves

The MIC of PEITC against *S. aureus* was assessed according to Miladi et al. [34] by using the micro broth dilution method. According to the previous study, the MIC of PEITC on *S. aureus* was around 0.6 mmol/L. The PEITC stock solution (1 mol/L) was added to TSB medium to achieve concentrations ranging from 0.0625 to 1 mmol/L. A sterile clear 96-well plate was supplemented with 100 μL of PEITC diluted in TSB medium at different concentrations and 5 μL of bacterial mixture at 1 × 10^6^ CFU/mL. The plate was incubated at 37 °C for 24 h, after which the absorbance of each well at 600 nm was determined by SpectraMax M2 (Molecular Devices, Silicon Valley, CA, USA). The lowest concentration of PEITC in the test wells, which did not significantly differ in absorbance from that in the blank control wells, was identified as the MIC.

To investigate the effects of PEITC on the growth curve of *S. aureus*, PEITC was added to TSB medium (100 mL) containing *S. aureus* (1 × 10^6^ CFU/mL), with concentrations ranging from 0.125 to 1 mmol/L. The cultures were incubated in Erlenmeyer flasks for 24 h (5000 rpm, 37 °C), and samples were taken every 2 h to measure absorbance at 600 nm.

### 2.3. Biofilm Formation

While the inhibition of biofilm formation in planktonic bacteria mainly involves interfering with bacterial adhesion and aggregation, the inhibition of already-formed biofilm, namely mature biofilm, primarily involves disintegration of the biofilm or direct action on the bacteria beneath the biofilm [35]. To determine the effect of PEITC on biofilm formation of planktonic *S. aureus*, the method of Bai et al. was used with minor modifications [19]. The bacteria were collected, centrifuged at 5000× *g*, and resuspended in TSB medium to 1 × 10^7^ CFU/mL. TSB medium (200 μL) containing PEITC at 0.25, 0.5 or 1 mmol/L was added to a 96-well plate. Similarly, to determine the effect of PEITC on mature biofilms, the wells were first filled with planktonic *S. aureus* (1 × 10^6^ CFU/mL) and incubated for 24 h to form a mature biofilm. A mixture of 200 μL of TSB solution and PEITC at 0.25, 0.5, and 1 mmol/L was then added to each well. After incubation for 12, 24, or 36 h, crystal violet solution (200 μL) was added, and the mixture was incubated for 15 min. Finally, the excess crystal violet stain was removed, 200 μL of methanol was added, and the absorbance at 595 nm was determined. The wells containing the bacterial mixture without PEITC were established as control wells. The residual biofilm in the wells was quantified by the previously described crystal violet method. The biofilm inhibition (destruction) rate was quantified as follows:

(1) $$\begin{array}{c} \mathrm{Inhibition}\;\left(\mathrm{destruction}\right)\;\mathrm{ratio}\;\left(\%\right)=\frac{\left({\mathrm{OD}}_{595}\;\mathrm{of}\;\mathrm{control}-{\mathrm{OD}}_{595}\;\mathrm{of}\;\mathrm{sample}\right)}{{\mathrm{OD}}_{595}\;\mathrm{of}\;\mathrm{control}}\times 100\% \end{array}$$

### 2.4. Protein Leakage

The protein leakage was assessed according to Kang et al., with modification [18]. To prepare the bacterial mixture, bacteria in the logarithmic growth phase were harvested by centrifugation, and the concentration of bacteria was adjusted to 1 × 10^7^ CFU/mL by TSB medium. PEITC was added to the bacteria at final concentrations of 0.25, 0.5, and 1 mmol/L. The cultures were incubated at 37 °C with agitation at 150 rpm for various durations, followed by centrifugation at 5000× *g* for 10 min. The supernatant was collected to determine protein leakage by a total protein assay kit (A045-4, Nanjing Jiancheng Bioengineering Institute, Nanjing, China). A group that did not receive PEITC treatment served as a control. The protein concentration was measured at 562 nm by using bovine serum protein as standard to form a standard curve (Y = 0.0085X + 0.0996, R^2^ = 0.9996), where Y and X represented the absorbance and protein concentration in μg/mL, respectively.

### 2.5. Total ATP Content

According to the previous study [18], the bacteria in the logarithmic growth phase were centrifuged at 5000× *g*, and the precipitates were resuspended and diluted to a concentration of 1 × 10^7^ CFU/mL using TSB culture medium. The final concentrations of PEITC at 0, 0.25, 0.5, and 1 mmol/L were subsequently added to the bacterial mixture, which was subsequently incubated at 37 °C for 1 and 4 h, respectively. After treatment, the bacteria were fragmented, and the total ATP content was then measured by an ATP kit (Beyotime Bioengineering Institute, Shanghai, China). The total ATP was expressed by relative luminescence unit (RLU).

### 2.6. ROS Production

ROS production was according to Li et al. [36]. The bacteria at 1 × 10^7^ CFU/mL were treated with 0, 0.25, 0.5, or 1 mmol/L PEITC. The samples were then incubated at 37 °C for 5 h for ROS determination. After treatment, the ROS fluorescent probe (DCFH-DA) was added, and the mixture was incubated for 30 min. The supernatant was collected by centrifugation (5000× *g*, 4 °C, 8 min), and the bacteria were resuspended in PBS. Fluorescence was observed by microscopy (Ti-2, Nikon, Tokyo, Japan). The average fluorescence intensity of the images was also quantified by ImageJ2. The amount of ROS in *S. aureus* was described by fluorescence intensity.

### 2.7. Scanning Electron Microscopy (SEM)

To create the mature biofilm model, coverslips were used as carriers. Coverslips were placed into a 6-well plate with bacteria and varying concentrations of PEITC and incubated for 24 h. The coverslips were then treated according to Fan et al. [37]; in brief, the coverslips were subsequently washed with 0.1 M phosphate buffer and fixed in 2.5% (*w*/*v*) glutaraldehyde (Sigma-Aldrich, Saint Louis, MO, USA) at 4 °C for 12 h. After two rinses with distilled water, the cells were dehydrated in a series of graded ethanol solutions (50%, 70%, 90%, and 100%) for 15 min each. The samples were then air-dried at room temperature and coated with a thin layer of gold–palladium, and the biofilms were visualized by SEM (JSM-7800-F, LEOL, Hokkaido, Japan).

### 2.8. Adherence of S. aureus

NCM460 cells (1 × 10^6^ cells/mL) in DMEM supplemented with 10% FBS and 1% Pen-Strep were incubated overnight (37 °C, 5% CO_2_). *S. aureus* in the logarithmic growth phase was separated into two groups. In one group, bacteria were centrifuged and resuspended in DMEM (1 × 10^8^ CFU/mL) and then incubated with the cells for 3 h (37 °C, 5% CO_2_). Each well was subsequently washed with PBS to remove unattached bacteria and lysed using 0.1% Triton X-100 (Beyotime Biotechnology, Shanghai, China), and colony counts were ascertained following serial dilutions [38]. Another group of bacteria was stained with SYTO™ 9, harvested by centrifugation, and subsequently cocultured with NCM460 cells. The unattached bacteria were removed by washing with PBS three times. Fluorescence inverse microscopy (Ti-2, Nikon, Japan) was used to capture images. The colony count value of each group was recorded as *A*_1_, and the colony count value of *S. aureus* at the time of inoculation was recorded as *A*_0_. The adhesion ratio was quantified as follows:

(2) $$Adhesion\;ratio\;\left(\%\right)=\frac{A_{1}}{A_{0}}\times 100\%$$

### 2.9. Quantitative Real-Time Polymerase Chain Reaction (qRT–PCR)

The bacteria at a concentration of 1 × 10^7^ CFU/mL were cultured at 37 °C for 24 h in the absence or presence of PEITC at 0.25 mmol/L. Total RNA extraction and purification were performed according to Wang et al. [28]; in brief, following the manufacturer’s protocol for the PrimeScript™ RT Kit with gDNA Eraser (TaKaRa, Otsu, Japan), RNA was modified to eliminate impurities and facilitate reverse transcription into cDNA templates. The 16S rRNA gene served as the internal reference, while specific primers for the differentially expressed genes identified through RNA-Seq were designed using Primer Premier 5.0 software and listed in Table 1. Amplification was performed in a 20 µL system by the SYBR Green Premix Pro Taq HS qPCR Kit (AG11701, Accurate Biology, Changsha, China). The differential gene expression level was evaluated by the 2^−∆∆Ct^ method, according to Ct value of each primer in different samples.

### 2.10. Application of PEITC in Beef Stored at 25 and 4 °C

According to a previous study with slight modifications [36], fresh raw beef was cut into 3.5 g cubes and sterilized, and each beef cube was placed into a sterile sampling bag. A bacterial suspension of *S. aureus* of 1 × 10^6^ CFU/mL (100 μL) was first inoculated into the sterile sampling bag to soak the beef, followed by the addition of 10 μL of various concentrations of PEITC (0.25, 0.5, or 1 mmol/L), which was diluted by TSB medium. The samples were then stored at 25 and 4 °C, respectively. The group without PEITC served as control. The number of living *S. aureus* in the beef were sampled at intervals of 0, 12, 24, 48, and 72 h. To quantify the viable count of *S. aureus* in the samples, 31.5 mL of sterile saline was added to a sterile sampling bag and homogenized (8 times/s, 60 s) using a sterile homogenizer (HBM-400B, Truelab, Shanghai, China). A 1 mL aliquot of the homogenized sample was subjected to gradient dilution from 10^1^ to 10^6^, 100 μL of the dilution was spread on a TSA plate and incubated at 37 °C for 24 h, and the total viable *S. aureus* on beef was calculated as log_10_ CFU/g after counting the colonies on each plate.

### 2.11. Statistical Analysis

The experiments were performed in triplicate, and the data are presented as the mean ± standard deviation. Statistical significance was ascertained through independent samples *t*-test, with a significance threshold set at α *=* 0.05.

## 3. Results and Discussion

### 3.1. Effects of PEITC on the Growth of S. aureus

**Figure 2:**
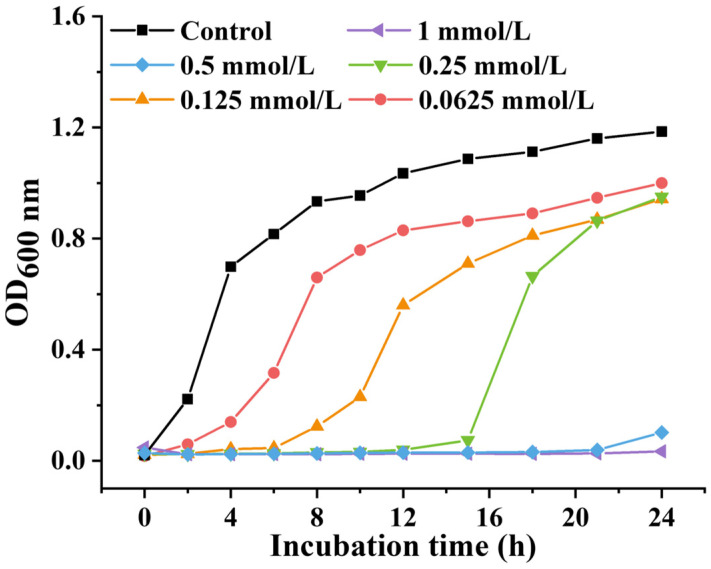
Growth curves of *S. aureus* under PEITC treatment.

To assess the inhibitory potential of PEITC against *S. aureus*, an MIC assay was conducted in a 96-well plate using the microdilution broth method. The MIC of PEITC against *S. aureus* was determined to be 1 mmol/L. The effects of different concentrations of PEITC on *S. aureus* were evaluated by bacterial growth curves using the shake flask method. As shown in Figure 2, bacterial growth was inhibited to various degrees by treatment with PIETC within 24 h. The time for complete inhibition of bacterial growth decreased with decreasing PEITC concentration from 0.125 to 0.5 mmol/L, and *S. aureus* began to grow at 6, 12, and 24 h, respectively. When the concentration of PEITC reached 1 mmol/L, the proliferation of *S. aureus* was entirely inhibited within 24 h. As the concentration of PEITC increased from 0.0625 to 0.5 mmol/L, the inhibition time of *S. aureus* growth ranged from 2 h to 21 h.

These results were consistent with those of a previous report [28], which demonstrated that, compared with other ITCs, especially BITC, PEITC had an equivalent inhibitory ability against *S. aureus*. When PEITC was applied to two different types of *E. coli*, the MIC was 0.40 mmol/L [39], and the MIC of PEITC against *P. aeruginosa* was 180.00 mmol/L [29], which was much greater than that of PEITC against *S. aureus* (1 mmol/L). These findings suggest that *S. aureus* is susceptible to PEITC.

### 3.2. Effects of PEITC on S. aureus Biofilm Formation

**Figure 3:**
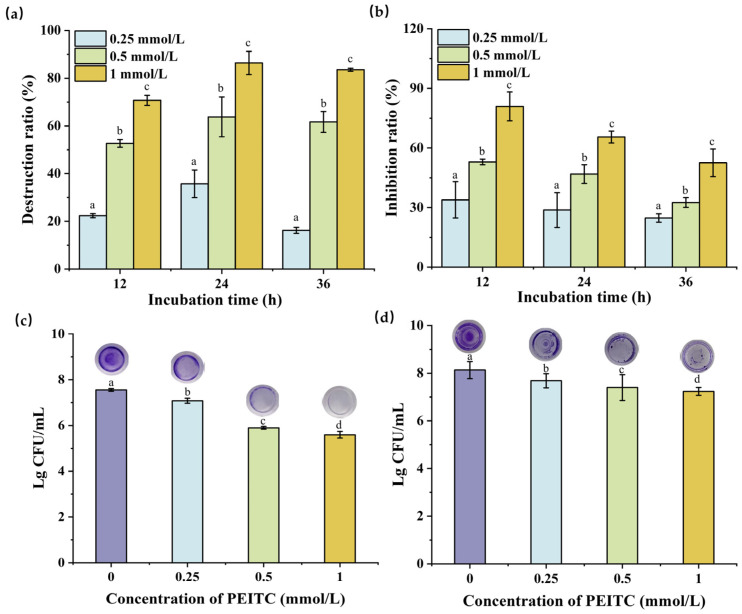
Effects of PEITC on *S. aureus* biofilms. Destructive effect on mature biofilms of *S. aureus* (**a**); Inhibitory effect on biofilm formation of *S. aureus* (**b**); Number of viable bacteria beneath the biofilms after treatment with PEITC for 24 h in the destruction process of biofilm (**c**); Number of viable bacteria beneath the biofilms after treatment with PEITC for 24 h in the inhibition process of biofilm (**d**); Different lowercase letters indicate a statistically significant difference between various concentrations (*p* ≤ 0.05).

The effect of PEITC on *S. aureus* biofilm formation is depicted in Figure 3. Overall, within 36 h, the mature biofilm of *S. aureus* was significantly disrupted by PEITC (*p* ≤ 0.05). Compared with the control, PEITC not only inhibited biofilm formation but also destroyed 86.42 ± 4.91% of the mature biofilm within 24 h. As the concentration of PEITC increased, the amount of disrupted biofilm gradually increased (Figure 3a). Over time, the disruptive effect of PEITC on mature biofilms initially increased but then diminished, peaking at 24 h. After 24 h of PEITC exposure, the biofilm disruption rates in the 0.5 and 1 mmol/L PEITC groups were significantly greater than those in the 0.25 mmol/L PEITC group, with disruption rates of 63.76 ± 8.34% and 86.42 ± 4.91%, respectively (*p* ≤ 0.05). Biofilms provide excellent protection to the bacteria they cover, and the number of viable bacteria beneath the biofilm is typically used to evaluate the residual amount of the biofilm. The living bacteria beneath the remaining biofilms after 24 h of treatment were determined (Figure 3c). For the mature biofilm, the destructive ability of PEITC increased as the concentration of PEITC increased. The inhibition ratio decreased gradually with time, and there was significant difference in the effects of diverse concentrations over the same durations (Figure 3b). At 12 h, the inhibition ratio of the 1 mmol/L PEITC treatment group was 80.90 ± 7.29%, indicating that PEITC significantly inhibited biofilm formation. The results shown in Figure 3d are consistent with those shown in Figure 3c, indicating that the effect on biofilms was proportional to the concentration of PEITC at the same incubation time. Combining the results shown in Figure 2, we extrapolated that the inhibition of *S. aureus* biofilms by PEITC is a synchronous process, where the disruption of the biofilm by PEITC occurs alongside the formation of new biofilm. Additionally, due to the difference in the initial bacterial load used for biofilm formation (10^7^ CFU/mL) compared to the MIC experiment (10^6^ CFU/mL), 0.5 mmol/L of PEITC was insufficient to completely inhibit biofilm formation. This explains the gradual decrease in biofilm disruption observed over time in Figure 3b. The disruption of mature biofilms is accompanied by the formation of new biofilms; thus, the optimal inhibition time for PEITC is 24 h.

The biofilms formed by *S. aureus* were a “shield” against antibacterial “spears”. They can provide robust protection to the bacteria beneath. Reducing biofilm formation is key to achieving *S. aureus* inhibition [40]. Several experiments have explored the influences of essential oils on biofilms. Allicin has been reported to significantly inhibit the formation of *S. aureus* biofilms, and the effect is enhanced by increasing the concentration of allicin from 0.39 to 3.90 mmol/L [41]. The inhibitory effect of allicin on *S. aureus* biofilms is similar to that of PEITC. When both allicin and PEITC are at MIC concentrations, biofilm production is only partially inhibited due to the different mechanisms of inhibition of bacterial growth and biofilm formation. However, the concentration of allicin to eliminate biofilm by 50% was 0.79 mmol/L in *S. aureus*, while PEITC can destroy 63.76 ± 8.34% of the biofilm at 0.5 mmol/L, indicating that PEITC exhibits a superior biofilm inhibition effect. Disruption of mature biofilms can also effectively reduce *S. aureus* contamination [42]. Substances capable of disrupting mature biofilms can exert more effective antibacterial action [43]. PEITC at 1 mmol/L almost completely disrupted existing mature biofilms after 24 h of treatment, thereby enhancing its antibacterial efficacy. According to previous reports, cardamom essential oil can effectively remove *S. aureus* biofilms at the lowest concentration of 0.125 mg/mL [44], which is much greater than the 0.04 mg/mL of PEITC (0.25 mmol/L) of PEITC. Therefore, we concluded that PEITC had a favorable inhibitory effect on *S. aureus* biofilm formation.

### 3.3. Effects of PEITC on Protein Leakage by S. aureus

**Figure 4:**
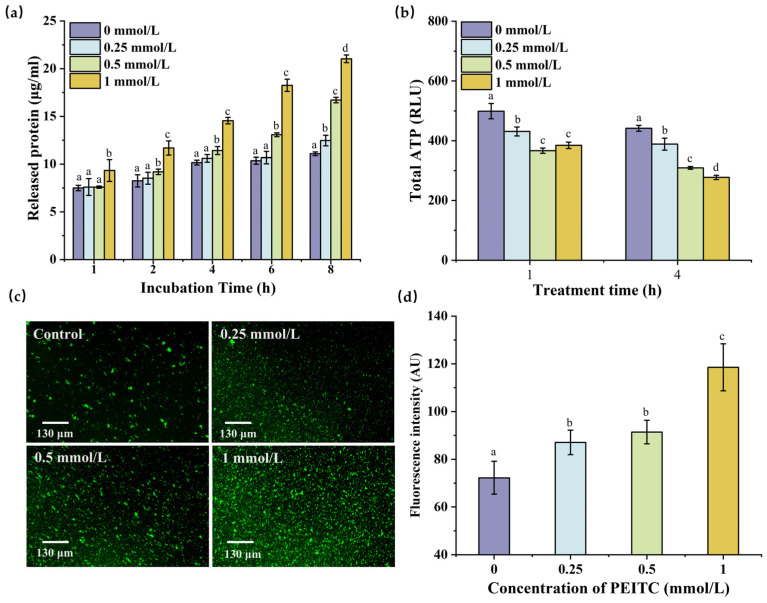
Effects of PEITC on the physiological metabolism of *S. aureus.* Protein leakage of *S. aureus* against PEITC (**a**); ATP content of *S. aureus* against PEITC (**b**); ROS produced by *S. aureus* against PEITC by SEM. The mean gray value was calculated by ImageJ (**c**,**d**). Different lowercase letters indicate a statistically significant difference between various concentrations (*p* ≤ 0.05).

The release of cytoplasmic contents is a widely recognized hallmark of bacterial membrane impairment [45]. Protein leakage was used to determine cell membrane integrity (Figure 4a), and the protein leakage in each treatment group gradually increased with time. When the PEITC concentration was 0.25 mmol/L, its significant impact on the protein leakage of *S. aureus* was only found at 8 h (*p* ≤ 0.05), indicating that 0.25 mmol/L of PEITC had a slight effect on the *S. aureus* membrane. As the concentration of PEITC gradually increased, the amount of protein leakage also increased. After incubation with 1 mmol/L PEITC for 8 h, *S. aureus* had the highest level of released protein (21.03 ± 0.42 μg/mL), up to 1.90 times that of the control group (*p* ≤ 0.05). These results indicated that PEITC at MIC significantly disrupted cell integrity.

The interference of antibacterial substances on bacterial membranes is an important index for evaluating the superiority of bacteriostatic substances. The addition of *Blumea balsamifera* (L.) DC. essential oils significantly increased the cell membrane integrity of *S. aureus* [46]. Kirazli et al. [47] reported that after treatment with nisin, the protein leakage of *S. aureus* increased to 14.12 ± 0.59 μg/mL; in comparison, the protein leakage caused by 1 mmol/L PEITC reached 21.03 ± 0.42 μg/mL. These results suggest that PEITC has a powerful destructive effect on the cytoplasmic membrane, causing intracellular substance efflux and negatively impacting the maintenance of the normal physiological functions of bacteria.

### 3.4. Effects of PEITC on Total ATP and ROS Production in S. aureus

Figure 4b shows the total ATP content in *S. aureus* treated with PEITC. The total ATP content decreased significantly from 1 to 4 h at all concentrations of PEITC, and that of the control group decreased from 499.07 ± 25.50 RLU to 441.33 ± 10.21 RLU. After treatment with different concentrations of PEITC, the total ATP content was markedly different from that observed in the control group (*p* ≤ 0.05). When *S. aureus* was incubated with1 mmol/L PEITC for 4 h, the total ATP content in *S. aureus* reached the lowest value of 277.19 ± 7.12 RLU, while the effect of PEITC decreased the total ATP in *E. coli* to approximately 400.31 ± 2.48 RLU [39]. This may indicate that total ATP production in *S. aureus* exhibits greater sensitivity to PEITC.

Excessive ROS can deform or even rupture bacteria [48]. To assess whether *S. aureus* undergoes oxidative stress in response to PEITC, to detect intracellular ROS levels, we utilized DCFH-DA, which can be oxidized by ROS to produce fluorescence. Upon excitation by a fluorescence beam, the fluorescence intensity of the bacterial cells was directly proportional to the ROS levels. After capturing the fluorescence images of the bacteria, ImageJ2 software was employed to quantify the ROS produced by *S. aureus*. As shown in Figure 4d, as the amount of PEITC increased, the green fluorescence progressively intensified, with the 1 mmol/L PEITC treatment group exhibiting the highest fluorescence. The ROS concentration reached 118.54 ± 9.83 AU, which was 1.6 times greater than that of the control group (*p* ≤ 0.05). However, compared to the group without PEITC, the groups treated with 0.25 and 0.5 mmol/L showed weak effects on fluorescence levels, with the mean gray values changing from 72.25 ± 6.90 AU to 87.07 ± 5.13 AU and 91.38 ± 4.95 AU, respectively.

The ATP and ROS production of *S. aureus* are closely related. When bacteria are subjected to certain external stresses, oxidative stress may be induced, leading to changes in the electron respiratory chain, resulting in increased ROS production [49]. A recent study concluded that daptomycin led to membrane stress and precipitated the generation of cytotoxic ROS [50]. PEITC may have a similar effect on *S. aureus* cell membranes and ROS production as daptomycin. The increased intracellular accumulation of ROS has detrimental effects on the cell membrane, thereby influencing the functionality of the electron respiratory chain, which is located within the cell membrane and is intricately associated with ATP production [51]. In our study, the elevation in ROS content was concomitant with an increase in PEITC concentration, which led to a decrease in the total ATP content, suggesting that excess PEITC may disturb physiological metabolism and impede the growth of *S. aureus.*

### 3.5. Effects of PEITC on the Morphology of S. aureus

**Figure 5:**
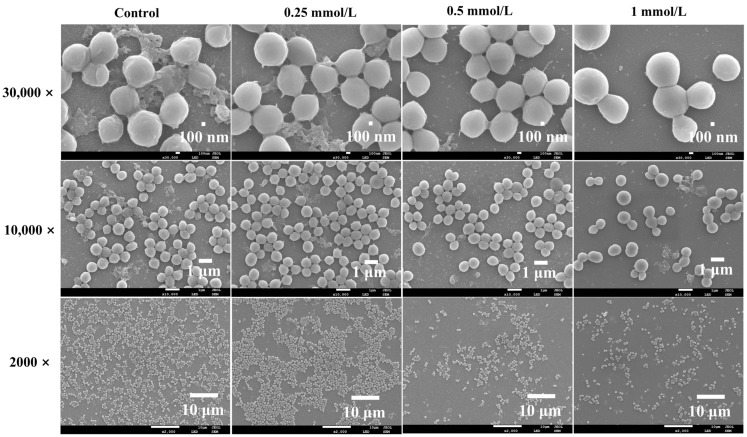
SEM images of the cell morphology, adhesion, and biofilms of *S. aureus* treated with PEITC.

We further explored the variations in the morphology of *S. aureus* treated with PEITC by SEM (Figure 5). As shown in the images at a magnification of 10,000×, clear variations were noted in *S. aureus* organisms after incubation with PEITC. The bacteria were smooth and uniform in size in the control group. As the concentration of PEITC gradually rose, the number of bacteria exhibiting significant morphological changes also increased. In the presence of 0.25 mmol/L PEITC, a few bacteria underwent significant swelling; as the PEITC concentration increased to 0.5 and 1 mmol/L, the bacteria became markedly larger, and rupture occurred. As shown in Figure 3c, PEITC at different concentrations can lead to a significant increase in ROS levels in *S. aureus,* and the accumulation of ROS can bring about changes in the fluidity and integrity of cell membrane [52]. In addition to the ability of PEITC to cause protein leakage in *S. aureus*, we further confirmed that PEITC could disrupt the robustness of the cell membrane. Nevertheless, the exact mechanism of PEITC action on the membrane is uncertain. Some essential oils can interact with fatty acids in the cell membrane directly [53]. Owing to the lipophilic nature of PEITC, it may easily penetrate the cell membrane, leading to bacterial rupture to a certain extent, disturbing *S. aureus* physiological metabolism and creating an imbalance of intracellular and extracellular osmotic pressures, which ultimately leads to the rupture of the bacterial bodies [54].

Notable differences in the biomass of biofilm were also observed at a magnification of 30,000×. The control group contained a substantial amount of biofilm, causing the bacterial cells to become rough due to biofilm encasement. As the concentration of PEITC increased, the amount of biofilm formed by bacterial cells gradually decreased. Although few biofilms were present under 0.25 mmol/L of PEITC, the amount of biofilm encapsulated around the bacteria was reduced, which made the surface of the bacteria smoother. As the concentration of PEITC reached the 0. 5 and 0.25 mmol/L levels, the biofilms on the surface of the bacteria were completely removed, and the architecture of the biofilm vanished. In the images magnified at 2000×, it can be observed that after being treated with PEITC for 24 h, the number of bacteria adhering to the slides gradually decreased. The number of bacteria remaining adhered to the slide in the presence of 0.5 or 1 mmol/L PEITC was less than 50.0% that of the control group. The mechanisms by which natural products inhibit the formation of *S. aureus* biofilms are diverse. They can interfere with the bacterium’s metabolism, hindering the secretion of polysaccharides and proteins, which are key components of the biofilm. For instance, polysaccharide intercellular adhesin (PIA), a significant polysaccharide, may also decrease under the pressure of natural antimicrobial agents, leading to weakened adhesion [55]. Bai et al. [19] utilized shikimic acid (SA) to assess its capacity to inhibit biofilms and reported that SA at 0.25, 0.5, and 1 mmol/L markedly diminished the biofilm biomass and adhesion of *S. aureus*, similarly to the effects of PEITC on *S. aureus*. A similar phenomenon occurred when *S. aureus* was treated with *Polygonum chinense* L. *aqueous* extract [56]. This may be because PEITC, as a small molecule, can penetrate the robust biofilm, inhibit the metabolism of *S. aureus*, and prevent planktonic *S. aureus* from adhering and aggregating. Overall, the SEM results, along with the findings in Figure 3 and Figure 4, confirmed that PEITC impacts the morphology of the bacteria, biofilm production, and adhesion of *S. aureus*.

### 3.6. Effects of PEITC on the Adhesion of S. aureus

**Figure 6:**
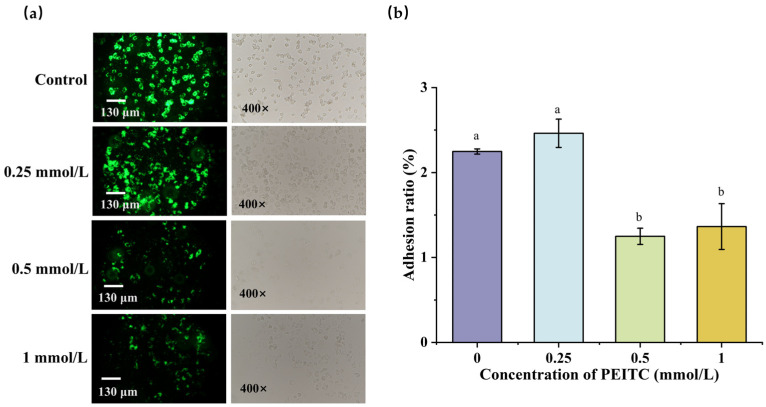
The effect of PEITC on the adhesion of *S. aureus*. Observation of *S. aureus* adhesion to NCM460 cells under fluorescence microscopy (**a**); Effect of PEITC on the adhesion ratio of *S. aureus* to NCM460 cells (**b**). Different lowercase letters indicate a statistically significant difference between various concentrations (*p* ≤ 0.05).

We evaluated the adhesion of *S. aureus* to NCM460 cells under the influence of PEITC. As shown in Figure 6a, the adhesion of *S. aureus* to NCM460 cells in the 0.5 and 1 mmol/L PEITC treatment groups was significantly weaker than that in the control group and the 0.25 mmol/L PEITC treatment group, as indicated by the decrease in fluorescence intensity around the cells. As shown in Figure 6b, we quantified the amount of *S. aureus* that adhered to NCM460 cells. No substantial distinction in adhesion was observed between the 0.25 mmol/L PEITC treatment group and the control group, whereas the adhesion rates in the 0.5 and 1 mmol/L PEITC treatment groups decreased by 44.38% and 39.34%, respectively. These outcomes reveal that PEITC can inhibit the adhesion of *S. aureus* to cells, potentially conferring some ability to counteract the intestinal invasion by *S. aureus* [20]. Similarly to PEITC, KLRE at a concentration of 64.00 mg/mL reduced the adhesion of *S. aureus* to the human lung carcinoma A549 cell line to about 50% compared to the positive control group [24]. To achieve a similar effect, a concentration of PEITC of 0.08 mg/mL (0.5 mmol/L) is required, much lower than that of KLRE. The ability of PEITC to inhibit the adhesion of *S. aureus* to NCM460 cells may stem from its impact on adhesion proteins, which are crucial for the bacterium’s attachment to epithelial cells. These bacterial surface proteins (including fibronectin-binding proteins) interact with specific cellular receptors or extracellular matrix components such as fibronectin activate signaling cascades that ultimately enable bacterial adhesion to host cells [57].

### 3.7. Relative Expression of Genes

**Figure 7:**
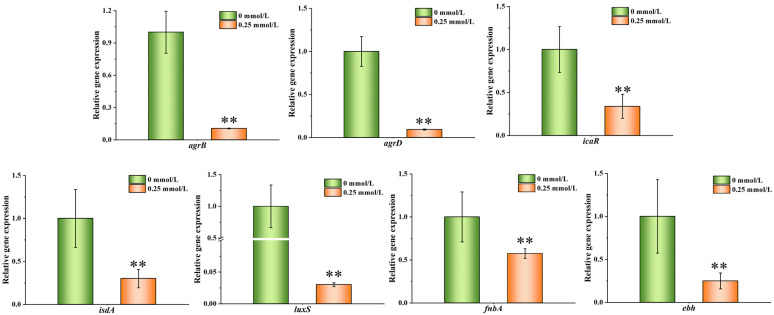
Relative expression of genes related to the adhesion and biofilm formation of *S. aureus* in response to PEITC. ** *p* ≤ 0.01, compared with control.

To determine whether PEITC can affect the expression of *S. aureus* genes, nine representative genes related to adhesion and biofilm formation were screened for qRT–PCR validation in 0.25 mmol/L PEITC (Figure 7). Compared with those in the control group, extremely significant decreases in *agrB*, *agrD*, *ebh*, *luxS*, *icaR*, *isdA*, and *fnbA* were detected (*p* ≤ 0.01). Among these, the most pronounced alteration was observed in *luxS*. Following exposure to 0.25 mmol/L PEITC, the relative expression level of *luxS* decreased to 3.04% of that in the control group. 

Research has shown that the virulence factors of *S. aureus* can damage host tissues, promoting bacterial colonization within the host. Most of these virulence factors are regulated by the auxiliary gene regulation (Agr) system, including *agrB* and *agrD*, which involves processes such as biofilm formation [58]. *LuxS* is also a potential target for local antibiotic therapy for biofilm association [59]. *IsdA* was the first protein shown to have physiologically relevant adhesion to both fibrinogen and fibronectin, which has a positive effect on *S. aureus* adhesion [60,61]. *FnbA* and *ebh* have both been revealed to play important roles in cellular adhesion [62]. All these genes were significantly downregulated under the influence of PEITC, which may lead to weakened adhesion, decreased resistance and slowed biofilm formation. These findings further suggested that PEITC has the potential to exert a comprehensive effect on *S. aureus* adhesion and biofilm formation by regulating related genes.

### 3.8. Effects of PEITC on Beef Stored at 25 and 4 °C

**Figure 8:**
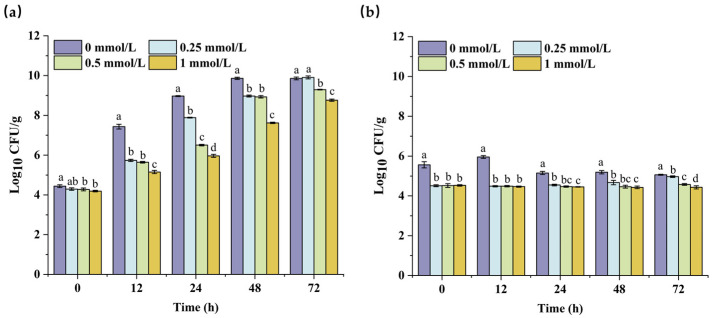
Application of PEITC to inhibit the growth of *S. aureus* in beef preserved at 25 °C (**a**) and 4 °C (**b**). Different lowercase letters on each time range indicate a statistically significant difference between various concentrations (*p* ≤ 0.05).

To investigate the efficacy of PEITC in controlling *S. aureus* in meat products, we established a beef model contaminated with *S. aureus*, as shown in Figure 8a. When beef was stored at 25 °C, the inhibitory effect of 0.25 mmol/L PEITC on *S. aureus* persisted for 48 h compared with that in the control group. However, after 72 h, the number of viable *S. aureus* bacteria in beef did not significantly differ from that in the control group, which can be attributed to the limited inhibitory effect of low-concentration PEITC on bacterial growth. As the concentration of PEITC increased to 0.5 and 1 mmol/L, the growth of *S. aureus* on beef was significantly slowed. After 24 h of storage, the concentration of *S. aureus* was 6.51 and 5.96 log_10_ CFU/g, respectively, compared to 8.97 log_10_ CFU/g in the control group. When the time reached 72 h, the concentration was 0.52 and 1.13 log_10_ CFU/g lower than that of the control group. These results indicated that with increasing concentration, the inhibitory capacity of PEITC against *S. aureus* in beef gradually increased, and the duration of its inhibitory effect increased over 72 h.

When the inoculated beef was stored at 4 °C (Figure 8b), the growth of bacteria in all the treatment groups slowed significantly. Compared with those of the control, the inhibitory effects of PEITC at diverse concentrations were significantly different. This may be due to the bactericidal action of PEITC against *S. aureus* contaminated beef, and the remaining *S. aureus* grew slowly at the low temperature of 4 °C. Unlike the inhibitory effect of PEITC on *S. aureus* at 25 °C, at 4 °C, not only did *S. aureus* fail to grow, but the total *S. aureus* count also decreased with storage time. This was the result of the combined effect of PEITC and low temperature. Under laboratory conditions, PEITC exhibited varying inhibitory effects on beef, which may be attributed to the heterogeneity in inhibitory concentrations arising from different cultivation environments and uneven distribution of PEITC [36].

## 4. Conclusions

In summary, PEITC strongly inhibited the physiological processes, biofilm formation, and adhesion of *S. aureus*. PEITC at a concentration of 1 mmol/L inhibited the growth of *S. aureus* for 24 h, significantly compromised the integrity of the cell membrane, and impeded ATP production. PEITC led to excessive ROS production in *S. aureus*, inducing an oxidative stress response, thereby affecting the proliferation of *S. aureus*. Furthermore, PEITC disrupted mature biofilms and inhibited new biofilm formation of the planktonic *S. aureus*. Additionally, PEITC not only reduced the adhesion and aggregation of *S. aureus* to carriers but also reduced the adhesion of *S. aureus* to NCM460 epithelial cells, indicating that it might have mitigating effects on *S. aureus* contamination of both mechanical surfaces and biological cells. Additionally, PEITC significantly downregulated genes related to *S. aureus* adhesion and biofilm formation. This suggested that the effect of PEITC on *S. aureus* was not merely a physical attack but also involved the weakening of *S. aureus* adhesion and biofilm formation abilities at the gene expression level. These combined actions enable PEITC to exhibit excellent antibacterial efficacy against *S. aureus*. The inhibitory effects observed in beef suggested that PEITC effectively inhibited the growth of *S. aureus* on beef. The whole study demonstrated that PEITC exerts a significant inhibitory effect on *S. aureus* biofilms and adhesion, and has inhibitory effects against *S. aureus* contaminated beef. As PEITC is a natural bioactive compound, the present study offers compelling evidence for the future application of PEITC and other bacterial inhibitors, particularly sulfur-containing compounds, in the food industry.

## Figures and Tables

**Table 1:** Sequences of Primers used for RT–qPCR.

Gene Name	Primer	Sequence (5′–3′)
*16 s rRNA*	*16 s rRNA*-F	CGTGCTACAATGGACAATACA
	*16 s rRNA*-R	ACAATCCGAACTGAGAACAAC
*isdA*	*isdA*-F	GTTGCAACAGCGAAATCTGA
	*isdA*-R	ATGCTTGTTTAGGCGTTTCG
*icaR*	*icaR*-F	TGCTTTCAAATACCAACTTTCAAG
	*icaR*-R	ACGTTCAATTATCTAATACGCCTGA
*fnbA*	*fnbA*-F	ATAGCGAAGCAGGTCACGTT
	*fnbA*-R	CCACCACCTGGGTTTGTATC
*luxS*	*luxS*-F	TAGATTAGCGGGAACGATGG
	*luxS*-R	ATGTAGTCCGGGCATATCCA
*ebh*	*ebh*-F	TTCCCAGCAGGTAATGGTTC
	*ebh*-R	GTTTTCGTAATCGGCGTTGT
*clfA*	*clfA*-F	TGCTGCACCTAAAACAGACG
*clfB*	*clfA*-R *clfB*-F *clfB*-R	TCCTGTTGTGCTGGATTTTG GCTGTTGCTGAACCGGTAGT GCCGCCATAAATGTGTTACC

## Data Availability

The original contributions presented in the study are included in the article. Further inquiries can be directed to the corresponding author.
